# Editorial: Influence of potential diagnostic biomarkers in lung cancer

**DOI:** 10.3389/fonc.2023.1271211

**Published:** 2023-08-15

**Authors:** Paul Takam Kamga

**Affiliations:** Université Paris-Saclay, UVSQ, EA4340 BECCOH, Boulogne-Billancourt, France

**Keywords:** lung cancer, diagnostic marker, NSCLC - Non small cell lung cancer, SCLC - small cell lung cancer, prognostic biomarker

Lung cancer remains the leading cause of cancer-related death worldwide (1). However, in recent years, there have been significant advancements in patient prognosis, primarily due to the discovery of a variety of therapeutic options. These include targeted therapies such as tyrosine kinase inhibitors (TKIs), immunotherapy with immune checkpoint inhibitors (ICIs), treatment combinations, and new treatment schedules for conventional therapies (2). The successful implementation of these treatment options is closely related to the precise molecular and cellular characterization of tumor samples. By identifying specific molecular aberrations, such as *EGFR, ALK, BRAF* mutations, or MET amplifications, oncologists can tailor treatments to target the underlying genetic alterations (3). Additionally, understanding the histological types, disease stages, metastatic spread, and PDL-1 levels plays a crucial role in devising personalized treatment plans. However, despite the advancements in treatment options, the prognosis for a significant fraction of lung cancer patients remains poor. This can be attributed to the insufficiency of available methods and or biomarkers to accurately diagnose, stratify, and predict patient responses to the available treatment options (2). Addressing these limitations in prognostic accuracy is crucial, as it can lead to improved screening efficiency, better risk stratification, and the ability to tailor treatments more effectively. Consequently, this has the potential to reduce health disparities among patients.

## 1 Optimizing the arsenal of imaging screening tools for lung cancer

The primary radiographical screening method for lung cancer remains LDCT (Low-Dose Computed Tomography), which has proven to be beneficial and indispensable. However, its implementation has resulted in only a modest 25% reduction in mortality (4–7). A significant drawback of LDCT is the occurrence of false-positive results, ranging from 60% to 94%, which can introduce biases in both diagnosis and treatment decisions (4). As a result, there is a growing focus on research to explore new diagnostic and imaging solutions that can complement LDCT or other conventional screening methods, including endomicroscopy during bronchoscopy. One promising approach involves the development of optical imaging using fluorescent probes, such as FAP-specific chemical probes, in combination with clinically compatible imaging systems. This innovative method can provide a readout of enzymatic activity, enabling disease monitoring, prognostication, and potentially aiding in therapy stratification (Mathieson et al.). Furthermore, the use of biological biomarkers like *EGFR* mutation status in conjunction with LDCT and/or other imaging methods such as 8F-fluorodeoxyglucose-positron emission tomography/computed tomography (18F-FDG-PET/CT)-based radiomics analyses showed promise in achieving both sensitive and specific early detection of NSCLC or predicting patients outcomes (Mathieson et al., Qi et al.).

## 2 Tissue biopsy in the era of precision medicine

Lung biopsies and samples from resectable surgery remain invaluable samples for screening and stratifying patients. They are utilized for histological and molecular characterization of the tumor bulk and the tumor microenvironment, thereby confirming the diagnosis and assessing disease progression (Qi et al., Gao et al.). Despite the challenges associated with obtaining these samples, researchers have made significant progress in refining the techniques used for their analysis. Advancements in minimally invasive biopsy procedures, such as endobronchial ultrasound-guided transbronchial needle aspiration (EBUS-TBNA) and image-guided percutaneous biopsies, have reduced the invasiveness and increased the feasibility of obtaining representative tissue samples (7). Furthermore, the development of cutting-edge screening methods, including highly sensitive immunohistochemistry (IHC) panels and advanced next-generation sequencing (NGS) technologies includingg DNA and mRNA sequencing on Formalin-fixed paraffin-embedded (FFPE) samples, has significantly improved the amount and quality of information that can be extracted from limited biopsy specimens. These techniques allow for the detection of specific biomarkers, genetic mutations, DNA methylation (Gao et al.) and expression patterns associated with diagnostic potential including ASCL1, NEUROD1, POU2F3, YAP1 (Gao et al.).

## 3 liquid biopsy in the era of precision medicine

In the quest for enhanced prognostic accuracy and personalized medicine, liquid biopsy has emerged as a revolutionary non-invasive technique in lung cancer management including SCLC and NSCLC (Adenocarcinoma, squamous cell carcinoma, and large cell carcinoma) (7, Peng et al., Wan et al.). It consists in the analysis of tumor-derived components in bodily fluids, such as whole blood and its derivative (plasma and serum), urine, saliva and cerebrospinal. Liquid biopsy offers a comprehensive and real-time assessment of genetic mutations and molecular alterations. Biomarkers analyzed through liquid biopsy as discussed by Kan et al. includes circulating tumor cells (CTCs) (Hong et al.), circulating tumor DNA (ctDNA) (Peng et al., Knapp et al.), circulating microRNA (miRNA), non-coding RNA (ncRNAs), and tumor-derived extracellular vesicles (EVs) (8). Most of these biomarkers originate directly from the tumor sites and enter the peripheral bloodstream during the early stages of the disease, making them indicators of early-stage cancer or potential markers for early metastasis. Accordingly CTC count or CTC aneuploid subtypes in the peripherepheral blood were investigated as acceptable biomarkers for diagnosing lung cancer, detecting early metastasis and predict patient survival (Xie et al., Zhang et al.). Expression level of signaling protein (chemokines/cytokines, ligands, receptors and mediators) represents a large field of liquid biopsy biomarkers that was addressed in this Research Topic. Tian et al. provided evidence that plasma CXCL14 may serve as diagnostic and prognostic biomarkers in lung cancer. Liu et al. used a lectin microarrays and blotting analysis to detect the differential expression of glycoproteins in bronchoalveolar lavage fluids (BALF) from a cohort of 281 patients with lung 281 patients with lung cancers. This study demonstrated that Protein Glycopatterns in BALF is Potential Biomarker for Diagnosis of Lung Cancer. The findings related to liquid biopsy and circulating biomarkers underscore the immense potential of this field, especially when coupled with robust analytical tools for genomic (NGS), proteomic (mass spectrometry, multiplexed Elisa, flow cytometry, etc.), and metabolomic analyses (Wan et al.). The use of these advanced analytical techniques has opened up new horizons (Wang et al.) and expanded the possibilities for early detection and characterization of various Lung cancer (Knapp et al.). Nevertheless, regarding the work of Gargiuli et al. on a cross-comparison of high-throughput platforms for circulating miRNA quantification, major challenges remain in the field. In fact, standardizing methods and defining expression cut-offs that hold clinical significance are crucial for the successful implementation of liquid biopsy biomarkers.

## 4 *In silico* methods and tools for biomarker discovery

A widely used tool in biomarker research is the in silico approach, which focuses on retrieving large datasets that have already been published and made available in well-known repositories, such as The Cancer Genome Atlas (TCGA) and the Surveillance, Epidemiology, and End Results (SEER) databases (9, 10). This approach allows researchers to gain initial insights into the potential value of biomarkers. Additionally, it can strengthen findings observed in small cohorts by analyzing larger and more diverse datasets. *In silico* assays were used in this issue to explore the prognostic value of KEAP1/NFE2L2 mutations in NSCLC and the diagnostic value of SHOX2 and RASSF1A DNA methylation in early lung adenocarcinoma (Gao et al., Zhu H. et al.). The integration of TCGA data with *in vitro* experiments proved fruitful in identifying the novel prognostic/diagnostic potential of HPGDS, whose expression was associated with lipid metabolism and aggressiveness in lung adenocarcinoma (Shao et al.). This underscores the importance of combining computational analyses with experimental approaches to comprehensively understand the significance of biomarkers in disease development and progression.

## 5 Functional studies

Functional studies indeed, play a critical role in understanding how diagnostic/prognostic biomarkers influence various lung cancer cell processes, including proliferation, differentiation, resistance to apoptosis, and drug response. In this Research Topic, studies employ a range of methodologies, such as loss and gain of function experiments, *in vitro analyses*, and *in vivo models*, to demonstrate that: i) Inhibitor of DNA binding 2 (ID2) functions is a tumor suppressor in lung adenocarcinoma (Chen et al.); ii) Choline Kinase Alpha2 promotes lipid droplet lipolysis in NSCLC, and its phosphorylation status correlates with a poor prognosis (Zhu R. et al.). By employing such functional studies, researchers gain valuable insights into the mechanisms through which specific biomarkers impact lung cancer biology, providing a foundation for potential therapeutic strategies and improved patient outcomes.

## 6 Conclusion

In summary this Research Topic comprehensively addresses the current challenges related to the development of potential and accurate diagnostic biomarker in Lung cancers. As technology continues to advance, the future of diagnostic/prognostic biomarkers holds great promise in transforming the landscape of medical research and patient care in lung cancer.

## Author contributions

PT: Writing – original draft, Writing – review & editing.
